# Long-Term Effects of Nitrogen and Lime Application on Plant–Microbial Interactions and Soil Carbon Stability in a Semi-Arid Grassland

**DOI:** 10.3390/plants14091302

**Published:** 2025-04-25

**Authors:** Kwenama Buthelezi, Nkosinomusa Buthelezi-Dube

**Affiliations:** School of Agricultural, Earth and Environmental Sciences, University of KwaZulu-Natal, P. Bag X01, Scottsville, Pietermaritzburg 3201, South Africa; kwenamahbuthelezi@gmail.com

**Keywords:** nitrogen fertilisation, plant stoichiometry, soil microbial activity, carbon sequestration

## Abstract

This study investigated the long-term (70 years) effects of N fertilisation (ammonium nitrate [AN], ammonium sulphate [AS]) at 70 and 211 kg N kg/ha, and liming (L) on plant–microbial interaction and soil carbon stability in a semi-arid grassland in South Africa. Aboveground biomass increased with N addition, particularly AN211, showing a 119% increase compared to the control, while both liming and N applications increased belowground biomass. Nitrogen addition significantly altered plant stoichiometric ratios, with root N ratios showing greater treatment-induced variation (12.7–51.3) than shoot N ratios (10.2–16.8). Microbial biomass carbon peaked with AN70 treatment, while dehydrogenase activity was highest in lime-only plots but suppressed in non-limed N treatments. Conversely, urease activity was highest in the control group and suppressed in most fertilised treatments. Despite increased biomass production, SOC remained unchanged across treatments (49.7–57.6 g/kg), whereas soil pH was lowest (<3.5) and highest (>6) under N fertilisation and lime, respectively. PCA revealed distinct clustering of treatments, with N forms differentially affecting plant allocation patterns and microbial parameters. This study demonstrates that plant–soil–microbe stoichiometric imbalances and pH-induced limitations on microbial function explain the disconnect between plant productivity and carbon sequestration in this semi-arid grassland ecosystem.

## 1. Introduction

Ecological stoichiometry theory provides a framework for understanding nutrient limitation and carbon cycling in grassland ecosystems [1,2]. This theory suggests that imbalances in elemental ratios (C:N:P) can constrain ecosystem processes through their effects on plant productivity, litter quality, and microbial decomposition [3]. In semi-arid grassland ecosystems, nitrogen (N) typically limits plant productivity [4], making these ecosystems particularly responsive to N addition, which is widely adopted to enhance biomass yields and overall C sequestration [5]. Long-term N inputs could exacerbate soil acidification, alter plant–microbe interactions, and disrupt carbon (C) sequestration pathways [6].

This study hypothesised that: (i) nitrogen and lime treatments would significantly alter plant tissue C:N:P ratios beyond ranges for microbial processing; and (ii) these stoichiometric shifts would correlate with changes in microbial biomass and enzyme activities that limit the incorporation of plant inputs into stable soil organic matter.

Nitrogen addition alters ecological stoichiometric balances [7,8], creating potential mismatches between plant inputs and microbial decomposition requirements. Plant C:N:P ratios regulate litter decomposition and microbial activity [9], yet the mechanistic links in plant stoichiometry, microbial function, and SOC persistence remain poorly understood, particularly under long-term management regimes. The response of plant and soil stoichiometry under N addition varies with N source, N application rate, and among ecosystems [10]. High N application rates, especially in ammonium form, often increase soil acidity, potentially suppressing microbial biomass [11,12]. However, differential effects of N sources (ammonium nitrate vs. ammonium sulphate) in mediating stoichiometric imbalances remain underexplored, particularly in long-term multi-decadal field experiments. Liming, while counteracting soil acidity, further complicates soil–plant–microbe interactions. By raising soil pH, lime can enhance microbial activity and root growth [13,14], yet long-term combined effects of liming and multiple N sources on plant–microbe stoichiometric coupling are rare. Soil enzyme activities, including urease and dehydrogenase, are important indicators of microbial functional responses to management practices and can provide insights into nutrient cycling dynamics [15]. Understanding the interplay between N sources, liming, and plant–microbial stoichiometric coupling is crucial for predictions of soil carbon stability under sustained management.

Most studies on grassland nutrient dynamics focus either on aboveground biomass or short-term responses, neglecting critical belowground feedbacks such as root C allocation, rhizodeposition, and extracellular enzyme activity [16]. While aboveground inputs have historically been considered the dominant source of C for soil and the primary control of SOC sequestration [17,18], recent evidence suggests that belowground inputs contribute more significantly to soil carbon stabilisation [16,19]. Understanding the long-term effects of N addition and liming on grassland ecosystems has significant implications in the context of global change. Rising atmospheric N deposition rates and increasing extreme events may interact with management practices to affect grassland carbon sequestration potential [20].

Decades of research in temperate grasslands, such as the Park Grass Experiment [21], have demonstrated that long-term N addition alters plant community composition, reduces biodiversity, and stabilises SOC through pH-mediated mechanisms. Similarly, the meta-analyses highlight consistent increases in aboveground biomass under N addition but reveal divergent SOC responses across ecosystems [12,22]. Yet, semi-arid grasslands remain underrepresented in these syntheses. Their unique biogeochemical conditions likely modify responses to fertilisation and liming, necessitating region-specific investigations. For example, prior work at the current study site revealed no changes in SOC after 70 years of N fertilisation and liming despite elevated plant productivity [11], suggesting complex interactions between nutrient management, plant allocation patterns, and soil processes.

This paper presents an integrated analysis of long-term nitrogen and lime treatments conducted at the Ukulinga research farm since 1951. By examining plant biomass allocation, stoichiometric ratios, nutrient uptake, and soil microbial parameters, this study aims to elucidate the mechanisms underlying the observed carbon dynamics in this semi-arid grassland system. We explore the central hypothesis that a long-term N and lime application creates stoichiometric imbalances in plant tissues that suppress microbial decomposition, explaining the disconnect between increased biomass inputs and SOC storage.

## 2. Results

### 2.1. Plant Biomass and Stoichiometric Responses to Long-Term N and Lime Application

Long-term nitrogen and lime application significantly affected plant biomass allocation and patterns (*p* < 0.05) (Table 1).

Values followed by a different lowercase letter in the same column are significantly different (*p* < 0.05) according to Tukey’s LSD procedure. C = control (0 kg/ha), L = lime (2250 kg/ha), AS70 = ammonium sulphate at 70 kg/ha; AS211 = ammonium sulphate at 211 kg/ha; AN70 = ammonium nitrate at 70 kg/ha; AN211 = ammonium nitrate at 211 kg/ha; AS70L = ammonium sulphate at 70 kg/ha + lime; AS211L = ammonium sul-phate at 211 kg/ha + lime; AN70L = ammonium nitrate at 70 kg/ha + lime; and AN211L = ammo-nium nitrate at 211 kg/ha + lime.

Shoot biomass was highest in AN211 (35,719 kg/ha) and AS70 (33,075 kg/ha), which were significantly greater than the control (16,324 kg/ha). The lime-only treatment and combination treatments did not significantly affect shoot biomass. Except for AN211, nitrogen application (AS70, AS211, and AN70) significantly increased root biomass compared to the control. Lime-only treatment also led to a significant increase in the root biomass (*p* < 0.01). However, its combination with N did not affect root biomass.

Only AN211 was significant for the shoot/root ratio amongst the N-only treatments. The AN211 treatment resulted in a significantly higher shoot/root ratio (48.11) compared to the control (19.26) and AN70 (6.56) (*p* < 0.001). On the contrary, lime-only treatment had a significantly lower shoot/root ratio (4.92) compared to the control (19.62). Except for AN70L, the combination of lime and nitrogen fertilisers had no significant effect on the shoot/root ratio. The shoot/root ratio under the AN70L (35.07) treatment was significantly higher than the control, AN211L (14.58), and L (4.92).

### 2.2. Plant Stoichiometric and Nutrient Uptake Responses to Long-Term N and Lime Application

**Figure 1:**
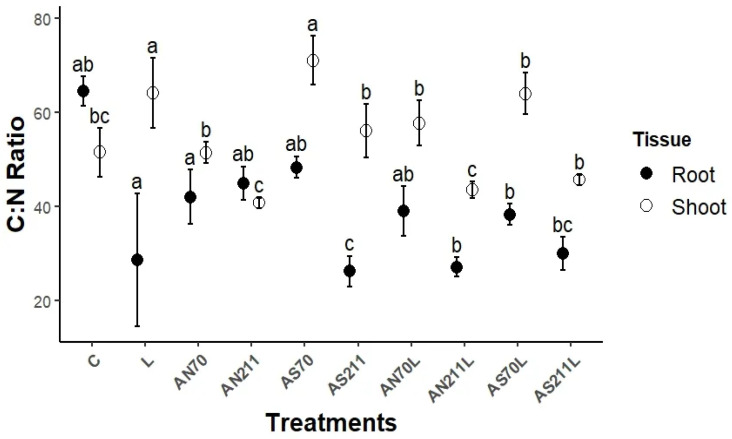
Response of the shoot and root C:N ratio to different liming and N additions. Means represented by same letter are not significantly different (*p* < 0.05) according to Tukey’s LSD procedure. C = control (0 kg/ha), L = lime (2250 kg/ha), AS70 = ammonium sulphate at 70 kg/ha; AS211 = ammonium sulphate at 211 kg/ha; AN70 = ammonium nitrate at 70 kg/ha; AN211 = ammonium nitrate at 211 kg/ha; AS70L = ammonium sulphate at 70 kg/ha + lime; AS211L = ammonium sulphate at 211 kg/ha + lime; AN70L = ammonium nitrate at 70 kg/ha + lime; and AN211L = ammonium nitrate at 211 kg/ha + lime.

The effect of liming and nitrogen application on elemental concentrations in plant tissues is available in Supplementary Table S1. Figure 1 shows that long-term nitrogen and lime application significantly altered shoots and roots’ C:N ratios. Only AS70 (71.2) and sole lime treatments significantly increased the shoot C:N ratio compared to the control (*p* < 0.05). Application of AN at 70 kg/ha significantly decreased the shoot C:N ratio compared to AN211 when applied alone (AN70) or in combination with lime (AN70L). The AS application rate was only significant amongst N-only treatments, where AS70 had a significantly higher shoot C:N ratio than AS211. The lime and nitrogen combination treatments had no significant effect on the shoot C:N ratio compared to the control.

Only AS211 was significant for the root C:N ratio, which was significantly lower (26.8) than the control (64.7) and other sole N treatments. The lime-only treatment had the most variable root C:N ratio, which was not significantly different from the control. The nitrogen application rate was not significant for the root C:N ratio for both AS and AN.

**Figure 2:**
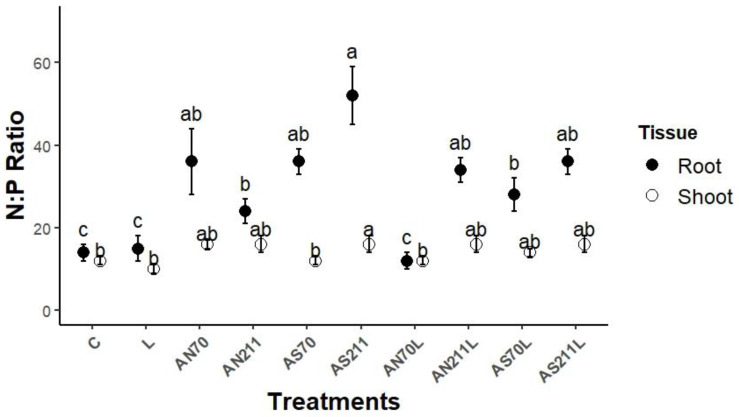
Responses of shoot and root N:P ratio in different treatments. Means represented by same letter are not significantly different (*p* < 0.05) according to Tukey’s LSD procedure. C = control (0 kg/ha), L = lime (2250 kg/ha), AS70 = ammonium sulphate at 70 kg/ha; AS211 = ammonium sulphate at 211 kg/ha; AN70 = ammonium nitrate at 70 kg/ha; AN211 = ammonium nitrate at 211 kg/ha; AS70L = ammonium sulphate at 70 kg/ha + lime; AS211L = ammonium sulphate at 211 kg/ha + lime; AN70L = ammonium nitrate at 70 kg/ha + lime; and AN211L = ammonium nitrate at 211 kg/ha + lime.

Long-term nitrogen and lime applications significantly altered plant tissue ratios (*p* < 0.001), with particularly pronounced effects on root tissues (Figure 2).

Root N:P ratios showed substantially higher values and significant treatment-induced variation compared to shoot N:P ratios. Root N:P ratios ranged from 12.7 to 51.3 across treatments, while shoot N:P ratios ranged from 10.2 to 16.8. All the nitrogen-only treatments had significantly higher root N:P ratios than the control. The nitrogen source effect was significant for 211 kg N/ha, where AS211 had a higher (51.3) root N:P ratio compared to AN211 (25.1). The effect of application rate was only observed for AS treatments, with AS211 having a significantly higher root N:P ratio than AS70 (36.7). Lime-only and AN70L treatments did not significantly affect the root N:P ratio (*p* > 0.05). However, AN70L significantly decreased the root N:P ratio compared to AN70 and AN211L. The AN211L, AS70L, and AS211L treatments led to significantly higher root N:P ratios than the control.

Only AS211 was significant for the shoot N:P ratio, which was higher than the control. No significant differences were observed between nitrogen sources for shoot N:P ratios at equivalent rates. However, shoot N:P ratios in nitrogen-treated plots were generally higher than in control and lime-only treatments. The nitrogen application rate was only significant for ammonium sulphate, with AS70 having a significantly lower shoot N:P ratio than AS211. The lime-only treatment was not significant for the shoot N:P ratio. Similarly, the treatments with a combination of lime and nitrogen had no significant effect on the shoot N:P ratio.

Only AN211 and AS211L significantly increased N uptake compared to the control (Supplementary Table S2). Phosphorus and K uptake were only significantly increased by AN211 treatment. Nutrient uptake followed the order N > K > P in these experiments.

### 2.3. Soil Properties and Microbial Responses

#### 2.3.1. Soil pH and SOC

**Figure 3:**
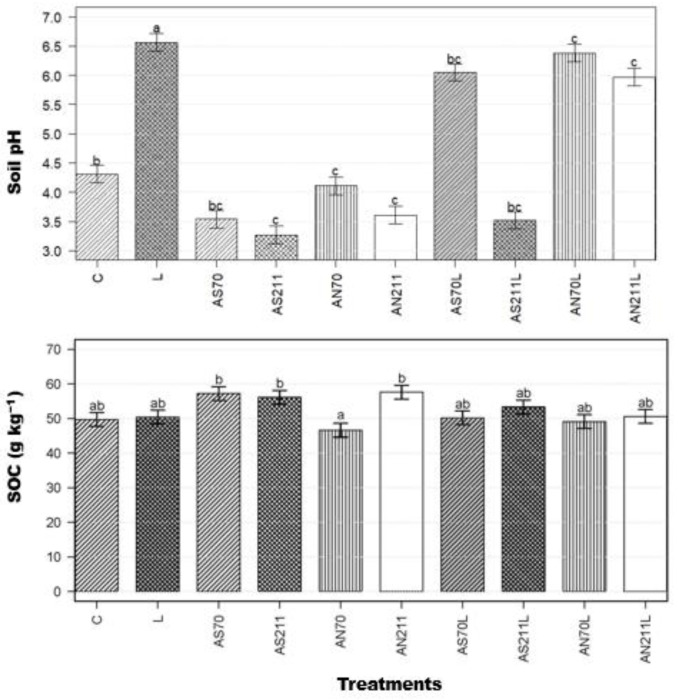
Soil pH and soil organic carbon after 70 years of lime and nitrogen addition (adapted from Buthelezi and Buthelezi-Dube, 2022). Means represented by same letter are not significantly different (*p* < 0.05) according to Tukey’s LSD procedure. C = control (0 kg/ha), L = lime (2250 kg/ha), AS70 = ammonium sulphate at 70 kg/ha; AS211 = ammonium sulphate at 211 kg/ha; AN70 = ammonium nitrate at 70 kg/ha; AN211 = ammonium nitrate at 211 kg/ha; AS70L = ammonium sulphate at 70 kg/ha + lime; AS211L = ammonium sulphate at 211 kg/ha + lime; AN70L = ammonium nitrate at 70 kg/ha + lime; and AN211L = ammonium nitrate at 211 kg/ha + lime.

Long-term nitrogen and lime application had a significant effect on soil pH (*p* < 0.001) but negligible changes were found in SOC (Figure 3).

Soil organic carbon ranged from 47.0 to 58.0 g/kg. Ammonium nitrate treatment at the lower rate (AN70) resulted in significantly lower SOC than the other nitrogen-only and N+L treatments. Except for AN70, nitrogen-only treatments significantly decreased soil pH (*p* < 0.01). Ammonium sulphate treatments without lime (AS70, AS211) resulted in low soil pH (3.54 and 3.27, respectively), which was not significantly different from AN211 (3.7). The lime-only treatment resulted in the highest soil pH (6.5) compared to the control (4.3). All AS and AN treatments with lime resulted in significantly higher soil pH than the control, except for AS211L.

#### 2.3.2. Microbial Biomass Carbon and Enzyme Activity

**Figure 4:**
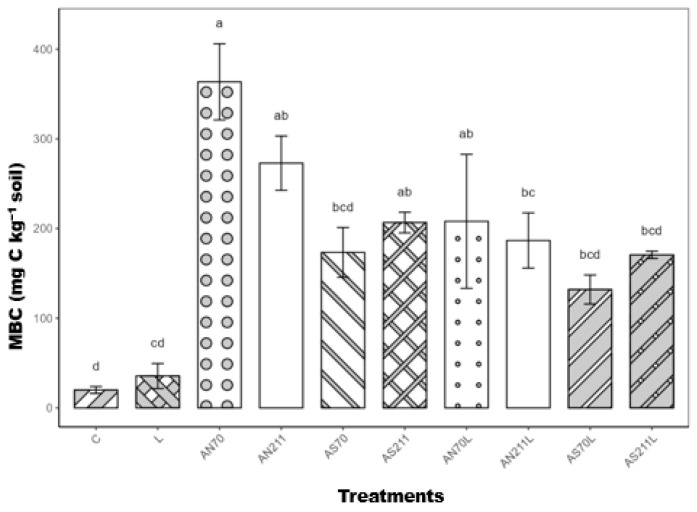
Microbial biomass carbon after 70 years of nitrogen and lime application. Means represented by same letter are not significantly different (*p* < 0.05) according to Tukey’s LSD procedure. C = control (0 kg/ha), L = lime (2250 kg/ha), AS70 = ammonium sulphate at 70 kg/ha; AS211 = ammonium sulphate at 211 kg/ha; AN70 = ammonium nitrate at 70 kg/ha; AN211 = ammonium nitrate at 211 kg/ha; AS70L = ammonium sulphate at 70 kg/ha + lime; AS211L = ammonium sulphate at 211 kg/ha + lime; AN70L = ammonium nitrate at 70 kg/ha + lime; and AN211L = ammonium nitrate at 211 kg/ha + lime.

Nitrogen fertilisation had a significant effect on MBC (*p* < 0.001) (Figure 4); however, the application rate was not significant. The AN70, AN211, and AS211 treatments significantly increased MBC compared to the control (*p* < 0.001). Plots with AS70, AS70L, AS211L, and L treatments had MBC that was comparable to the control.

**Figure 5:**
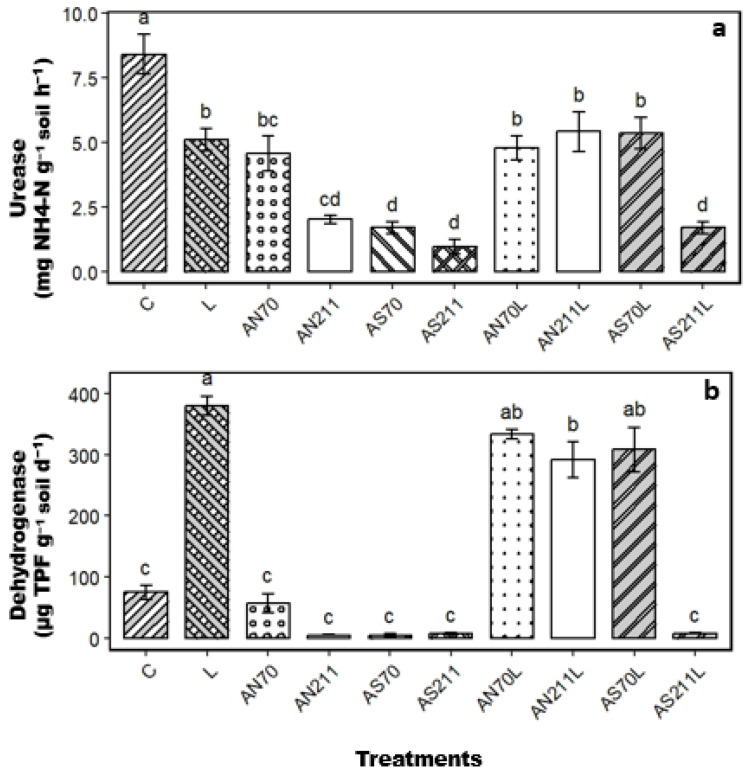
Urease (**a**) and dehydrogenase (**b**) activity following 70 years of nitrogen and lime application. Means represented by same letter are not significantly different (*p* < 0.05) according to Tukey’s LSD procedure. C = control (0 kg/ha), L = lime (2250 kg/ha), AS70 = ammonium sulphate at 70 kg/ha; AS211 = ammonium sulphate at 211 kg/ha; AN70 = ammonium nitrate at 70 kg/ha; AN211 = ammonium nitrate at 211 kg/ha; AS70L = ammonium sulphate at 70 kg/ha + lime; AS211L = ammonium sulphate at 211 kg/ha + lime; AN70L = ammonium nitrate at 70 kg/ha + lime; and AN211L = ammonium nitrate at 211 kg/ha + lime.

Nitrogen fertilisation as ammonium nitrate (AN) significantly increased urease activity (*p* < 0.001), while ammonium sulphate resulted in no change (Figure 5a). The rate of nitrogen fertiliser was not significant for both AN and AS. Lime-only treatment led to a significant increase in urease activity. Similarly, plots with lime + N treatments (AN70L, AN 211L, and AS70L) had significantly higher urease activity. The AS211L treatment had comparable urease activity to the control.

Dehydrogenase activity significantly varied across treatments (Figure 5b). Nitrogen-only treatments had no significant effect on dehydrogenase activity, and the AS211L treatment also had no significant effect. However, AN70L, AN211L, and AS70L had significantly higher dehydrogenase activity comparable to the L treatment.

#### 2.3.3. Multivariate Analysis of Plant, Soil, and Microbial Properties

Correlation matrix results (Supplementary Figure S1) showed a strong positive correlation between shoot biomass and total plant carbon (1:00), soil pH, and dehydrogenase (0.98), while urease had moderate to strong negative correlations with several variables: soil P (−0.70), shoot biomass (−0.59), and total plant C (−0.62). Soil P showed positive correlations with shoot biomass (0.63) and total plant C (0.64). SOC correlated positively with soil P (0.62), and MBC had a negative correlation with shoot biomass (−0.47) and total plant C (−0.37). The root/shoot ratio (shoot C:N ratio) shows little correlation with plant biomass variables, but moderate correlation with MBC (0.42). Root biomass shows weak correlation with most variables, with its strongest relationship being a negative correlation (−0.36) with soil pH and dehydrogenase.

**Figure 6:**
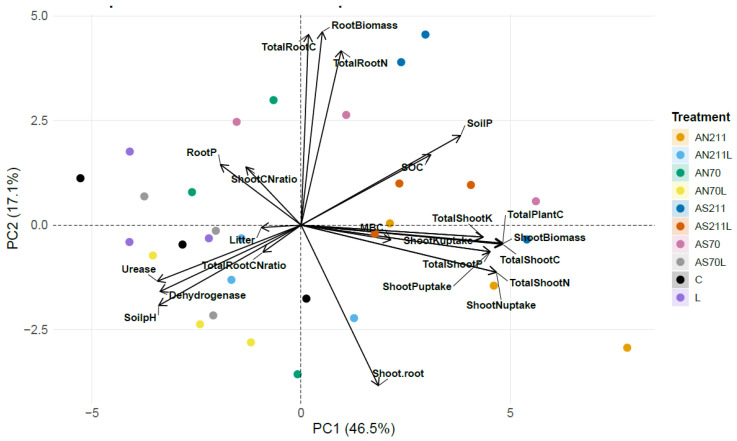
Principal component analysis biplot showing relationships between plant, soil, and microbial parameters after 70 years of lime and nitrogen application. C = control (0 kg/ha), L = lime (2250 kg/ha), AS70 = ammonium sulphate at 70 kg/ha; AS211 = ammonium sulphate at 211 kg/ha; AN70 = ammonium nitrate at 70 kg/ha; AN211 = ammonium nitrate at 211 kg/ha; AS70L = ammonium sulphate at 70 kg/ha + lime; AS211L = ammonium sulphate at 211 kg/ha + lime; AN70L = ammonium nitrate at 70 kg/ha + lime; and AN211L = ammonium nitrate at 211 kg/ha + lime.

The principal component analysis biplot (Figure 6) revealed distinct patterns among treatments and measured parameters, with PC1 and PC2 explaining 46.5% and 17.1% of the total variance, respectively.

PC1 was primarily characterised by a positive loading of plant biomass parameters including shoot biomass, total plant C, shoot K and N content and uptake. In contrast, soil parameters such as soil pH, enzyme activity loaded negatively on PC1. PC2 had positive loading for shoot/root ratio and soil parameters (MBC, soil pH and dehydrogenase), while the root biomass loaded negatively on this axis (Figure 6).

The biplot with 95% confidence ellipses revealed distinct clustering of treatment groups. The ammonium nitrate [AN] treatments (AN70; AN211) generally clustered towards the positive PC1 axis while ammonium sulphate [AS] treatments (AS70; AS211) showed intermediate positioning between the control and AN treatments. The lime-only treatment is positioned towards the negative PC1 axis while N + lime treatments (AN70L, AN211L, AS70L, AS211L) demonstrated a shift along PC2 compared to other non-limed counterparts. The control occupied the centr position, primarily along the negative side of PC1. The distinct separation of confidence ellipses among treatments demonstrate that the N fertilisation and liming resulted in significantly different plant-soil-microbial parameter profiles.

## 3. Discussion

Nitrogen application facilitates aboveground growth, which alters shoot/root ratios in grassland ecosystems [23]. The current findings demonstrate that the response to nitrogen treatments at the Ukulinga grassland varied considerably depending on the form and rate of nitrogen application. The significant increase in aboveground production by AS70 (33.075 kg/ha) and AN211 (35.72 kg/ha) treatments compared to the control (16.32 kg/ha) indicates that nitrogen availability is a primary limiting factor in this ecosystem. This is consistent with previous studies reporting significant increases in aboveground biomass following nitrogen addition [12,22]. The remarkably high shoot/root ratio observed in AN211 plots (48.11) compared to all other treatments suggests an extreme allocation of resources to aboveground production at the expense of root development. This is consistent with the trend reported in a recent meta-analysis, which showed that N addition leads to more biomass [22].

At lower application rates (70 kg/ha), ammonium sulphate (AS70) produced significantly higher shoot biomass than ammonium nitrate (AN70), supporting earlier findings by [24] at this same experimental site. However, at higher application rates, ammonium nitrate (AN211; 35.72 kg/ha) had significantly greater shoot biomass than ammonium sulphate (AS2110; 23.80 kg/ha). This reversal reflects a physiological threshold where excessive NH4+ becomes toxic to plants, while NO3- remains beneficial at higher concentrations [25]. The NH4+ toxicity would have been more pronounced due to significant soil acidification (3.27–3.54) when ammonium sulphate (Figure 3) is applied, resulting in immediate growth restriction [26]. Applying ammonium sulphate results in a more acidic rhizosphere than ammonium nitrate because only 50% of AN-N is in the NH4+ -N form compared to 100% in the AS fertiliser [27]. Consequently, soil pH (Figure 3) was significantly low (<3.5) in plots with ammonium sulphate treatments (AS70, AS211), while lime and lime + nitrogen treatments (AN70L, AN211L) resulted in the highest pH values (>6.0). In contrast, lime treatments significantly reduced the shoot/root ratio to 4.92, primarily by stimulating root growth (259 kg/ha) while suppressing shoot production (12.74 kg/ha) compared to the control. The lack of a positive response to separate lime-only application in shoot biomass (actually showing a reduction compared to the control) (Table 1) potentially reflects phosphorus deficiency, as reported by [11], who found low extractable P in these limed plots. This suggests that N and P are limiting factors for plant productivity in the Ukulinga grassland. However, the positive response in root biomass to liming indicates a shift in resource allocation towards belowground organs under these conditions. When lime was combined with ammonium sulphate (AS70L and AS211L), it moderated the negative effects on shoot biomass observed in the nitrogen-only treatments while maintaining moderate root development. However, when combined with ammonium nitrate, particularly at a lower rate (AN70L), it substantially reduced both shoot biomass (12.36 kg/ha) and root biomass (38.1 kg/ha), resulting in the lowest root production among all treatments but maintaining a high shoot/root ratio (35.07). These pH differences are thus crucial for understanding plant–soil interactions and microbial processes.

Elemental composition (Supplementary Table S1) revealed significant effects on tissue C, N, and P concentrations, further supporting the observation of altered resource allocation. Treatments significantly affected aboveground nitrogen concentrations, with nitrogen treatments showing the highest values. The belowground nitrogen concentration was also significantly affected by treatments, with AN211L showing the highest value (1.40%), supporting narrower C:N ratios in fertilised plots. This elemental composition confirms the stoichiometric shifts discussed below. The lack of response to shoot P concentrations combined with greater shoot biomass may indicate that more efficient utilization of P is occurring. The phosphorus allocation pattern is driven by soil P availability and affects leaf photosynthetic rates of plant species [28]. Similar to other studies [9], P concentration in roots differed more across treatments than N. Long-term application of N at Ukulinga grassland has decreased root P concentration, consistent with results shown by a meta-analysis [29].

The results prove the hypothesis that nitrogen and lime treatments would significantly alter plant tissue C:N:P ratios beyond ranges for microbial processing. The N:P ratios in fertilised plots generally increased to values greater than 14, indicating a co-limitation of N and P, while the control and lime treatments showed values less than 10, suggesting N limitation [9,30] (Figure 2). The stoichiometric imbalance likely constrained belowground C allocation and created potential mismatches with microbial stoichiometric requirements. This is further supported by higher N uptake in the AN211 treatment (415.7 kg/ha) compared to the control (140.6 kg/ha) without proportional increases in P uptake, thus creating wider N:P ratios.

Nitrogen fertilisation treatments showed varied effects on tissue C:N ratios. AS211 treatments significantly reduced the root C:N ratio compared to the control (Figure 1). Interestingly, AN70L and AN211L treatments also substantially reduced root C:N ratios, a trend observed for all fertilisation treatments, while the shoot C:N ratios were more variable. This indicates a stronger effect of nutrient management on belowground than aboveground tissue quality, which has important implications for decomposition and soil carbon dynamics. The control plots exhibited a higher root C:N ratio compared to the shoot C:N ratio, while the lime-only treatment significantly reduced the root C:N ratio, with the shoot C:N ratio remaining relatively high (Figure 1). This suggests enhanced N concentration in root tissues under liming, potentially making them more decomposable by microbial communities. While shoots generally maintained higher C:N ratios (ranging from 40 **to** 70) across treatments, roots showed greater sensitivity to treatments, with C:N ratios varying from 27 to 65). The observed differences in C:N ratios between shoots and roots across treatments highlight the distinct nutrient economies of above- and belowground plant components. Furthermore, the results suggest that root tissue quality may be a more responsive indicator of management effects. These findings suggest that semi-arid grasslands could experience amplified stoichiometric imbalances, particularly under projected N deposition rates, which could impair microbial decomposition and nutrient cycling.

The reduced root C:N ratios in fertilised and limed plots would theoretically enhance rather than limit microbial decomposition, as narrower C:N ratios typically favour microbial activity [31]. However, our results suggest that the C:N ratio is not the dominant predictor of decomposition rates as soil organic carbon did not increase (Figure 3) after 70 years of N fertilisation and liming. There may be a disconnect between plant tissue quality and carbon accumulation, with observed stoichiometric imbalances likely constraining belowground C allocation, decoupling biomass production from SOC accrual. Extreme soil acidification (pH < 3.5) in ammonium sulphate treatments likely inhibited microbial decomposers, aligning with pH-driven C stabilisation mechanisms reported by [32] and supported by the microbial and enzyme activity data (Figure 5 and Figure 6). The lack of SOC response can also be explained by the following mechanisms. Firstly, priming effects may have offset potential gains from increased plant inputs [33] due to high nutrient availability. Studies have observed that under conditions of high nutrient availability, microbes may switch from decomposing recalcitrant soil organic matter to using labile root exudates for their carbon and energy requirements [34,35]. Secondly, observed stoichiometric imbalances in plant tissues, particularly increased N:P ratios beyond optimal ranges for microbial processing, may have created a functional mismatch between plant inputs and microbial decomposer requirements. Lastly, given the high soil C (49.7–57.6 g/kg across treatments), soil physical protection mechanisms may have reached saturation in this soil, thus limiting stabilisation regardless of input quantity. Similar to the results of [6,36], N fertilisation increased MBC, with the AN70 treatment resulting in the highest MBC (>350 mg C/kg soil) compared to the control and limed treatments (< 50 mg C/kg soil). Nitrogen from synthetic fertilisers is readily available to generally N-limited microbes, explaining the significant increase in MBC in N treatments [37].

Urease and dehydrogenase showed a significant response to treatments, with the control plots having the highest urease activity (Figure 5a), while lime substantially increased dehydrogenase (Figure 5b) and decreased MBC. This is consistent with the results of [15,38], who also recorded a similar response of MBC and dehydrogenase to lime application. The contrasting responses of MBC and enzyme activities across treatments suggest significant shifts in microbial community composition and function beyond simple changes in biomass. In N-treated plots, suppressing dehydrogenase activity despite moderate MBC indicates potential shifts from bacterial to fungal dominance, as fungi generally tolerate acidity better than bacteria [39]. Recent studies have demonstrated that N addition can significantly after soil microbial community composition in grasslands, with potential consequences for decomposition processes and carbon storage [40]. Moreover, the increase in dehydrogenase in limed plots may suggest the availability of more easily decomposable components of crop residues for the metabolism of soil microorganisms [41]. While these measured microbial parameters provide valuable functional information about microbial response to long-term treatments, they do not capture the full complexity of the microbial community composition and structure. Modern metagenomic approaches would be valuable in confirming these hypothesised community shifts and their functional implications for carbon cycling. These enzyme patterns provide critical insights into the disconnect between increased plant inputs and SOC accumulation. Despite high biomass production in fertilised plots, the suppression of dehydrogenase activity under acidic conditions (pH < 3.5) in these plots indicates impaired microbial capacity to process inputs into stable SOC fractions. The differential responses of MBC and enzyme activities across treatments suggest that N addition altered not just microbial biomass but also their functional capacity.

The AN211 treatment resulted in significantly higher N uptake (415.7 kg/ha) than all other treatments, more than doubling the control (140. 6 kg/ha) (Supplementary Table S2). This supports the observation that nitrate is the preferred form of nitrogen uptake at higher application rates. Moreover, increased N availability upon N addition increases N absorption via roots, increasing foliar N concentration [32]. Plant tissue N concentration can also be affected by plant-specific N use strategies and efficiency [36]. Similarly, P and K uptake were highest in AN211 (26.55 kg/ha and 335 kg/ha, respectively), suggesting efficient nutrient utilization.

The PCA (Figure 6) and correlation (Supplementary Figure S1) analyses reveal the fundamental mechanistic relationships driving the ecosystem responses to treatments. Shoot biomass is a primary contributor to total plant carbon, supported by a strong correlation between these two variables. The negative correlation between soil P and urease suggests that urease activity is associated with lower P availability and reduced plant growth, as supported by biomass data and urease activity (Table 1 and Figure 5a). The negative correlation between MBC and plant variables suggests a possible competition between microbes and plants for resources. The shoot C:N ratio correlated with MBC, suggesting that different allocation patterns may be linked to soil microbial communities.

PC1 primarily represents a gradient from high pH and lower biomass conditions (negative scores) to higher biomass and lower pH conditions (positive scores), re-emphasizing the acidifying effects of nitrogen fertilisation on plant growth. Moreover, PC1, demonstrates a clear separation between management strategies that favour either aboveground or below-ground processes. The strong association of N-only treatments (especially AN211) with shoot parameters (biomass, C, N and uptake) and MBC suggests that these treatments enhance photosynthetic capacity and carbon flow primarily to shoots and subsequently to microbial communities through rhizodeposition. This is supported by the clustering of ammonium treatments towards the positive PC1 axis corresponding with high plant biomass and nutrient uptake (Table 1 and Supplementary Table S2).

The shift of N treatments with lime along PC2 compared to their non-limed counterparts indicates altered resource allocation patterns between shoots and roots. Conversely, lime treatments establish a distinct functional domain characterised by higher soil pH, enhancing enzyme activity (particularly dehydrogenase), and greater root development. This suggests that liming fundamentally alters carbon allocation pathways by promoting root exploration and creating soil chemical conditions favouring specific functional groups. The apparent clustering of treatments in the PCA confirms that management practices create distinct biogeochemical regimes within the grassland ecosystem, with implications for carbon and nutrient cycling beyond simple productivity metrics.

Our findings have important implications for understanding ecosystem responses to global nitrogen deposition. Despite current global deposition rates (10–20 kg N/ha/yr) being lower than our experimental rates, these are projected to increase in many regions [42]. Observed stoichiometric imbalances and soil acidification under high N inputs may foreshadow long-term consequences of chronic atmospheric N deposition. Meta-analyses of global N addition experiments and soil acidification have shown variable effects on soil carbon storage [43], with some studies reporting positive effects in N-limited systems and others showing neutral or negative effects in systems receiving higher background N deposition. Contrary to the current findings, ref. [44] showed substantial increases in soil carbon in some fertilised Park Grass plots. This could be explained by contrasting climate conditions, with higher rainfall and cooler temperatures at Park Grass promoting greater carbon stabilisation compared to the current semi-arid site. However, consistent with this study’s observation of altered shoot ratios under high N treatments, ref. [45] found that N addition increased aboveground productivity but reduced belowground biomass allocation. The current results suggest that soil pH changes may be a critical mediating factor explaining this variability. Systems with higher buffering capacity may maintain favourable conditions for microbial processing of increased plant inputs and carbon stabilisation under N deposition, while poorly buffered systems could experience acidification that limits microbial decomposition.

It is important to note that the sampling depth considered in this study (0–10 cm) presents a limitation. This shallow sampling depth was chosen because this zone represents the grassland root-mat where approximately 60–80% of grass root biomass is concentrated [46]. In addition, the majority of soil organic matter accumulation, nutrient cycling, and microbial activity occurs in the topsoil layer due to the concentration of fine roots and microbial communities [47]. Additionally, the shallow sampling depth minimises site disturbance in this long-term experimental plot, preserving the integrity of ongoing research.

While deeper soil layers (>10 cm) can contribute significantly to carbon storage in some ecosystems, previous studies in semi-arid grasslands have shown that management-induced changes in soil carbon are most pronounces in the top 10 cm [8]. However, future research should incorporate deeper sampling depths to fully account for potential management effects on subsoil carbon dynamics.

## 4. Materials and Methods

### 4.1. Study Site

**Figure 7:**
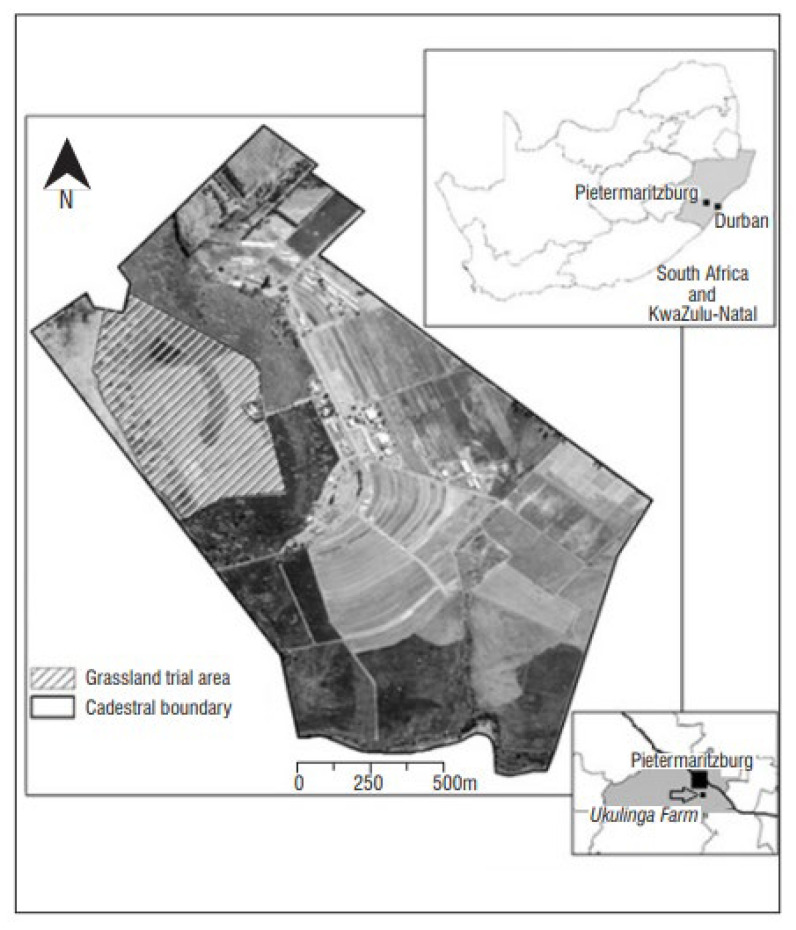
Location of Ukulinga grassland experiment site [48].

The study was done in a long-term grassland trial located at Ukulinga, a research farm of the University of KwaZulu-Natal in Pietermaritzburg, South Africa (29°24′ E, 30°24′ S) (Figure 7).

The region is semi-arid, with an average annual precipitation of 790 mm, and is situated on a plateau at 838 m above sea level. It features warm summers, with a mean monthly maximum temperature of 26.4 °C in February, and mild winters, averaging 3.2 °C in July, with occasional frost. The soil originates from localized dolerite intrusions into a shale parent material. The area is underlain by shale with localised dolerite intrusions with the vegetation is characterized by southern tall grass veld or, on a broader scale, KwaZulu-Natal hinterland thornveld, which is an open savanna dominated by Acacia (Vachellia) sieberiana with patches of *Hyparrhenia hirta* L. and other herbaceous species [49].

### 4.2. Experimental Design

The long-term veld fertiliser experiment trial started in 1951 with the application of nitrogen, phosphorus, and lime. The experiment was laid out in a randomized block design with 9.0 m × 2.7 m size plots replicated three times. This study focused on nitrogen applied annually as ammonium nitrate and ammonium sulphate at 70 and 211 kg N/ha and lime applied as dolomite at 2250 kg/ha every five years. The following ten treatments were selected: (1) control (0 lime or N fertiliser kg/ha), (2) L = lime (2250 kg/ha), (3) AS70 = ammonium sulphate at 70 kg/ha; (4) AS211 = ammonium sulphate at 211 kg/ha; (5) AN70 = ammonium nitrate at 70 kg/ha; (6) AN211 = ammonium nitrate at 211 kg/ha; (7) AS70L = ammonium sulphate at 70 kg/ha + lime; (8) AS211L = ammonium sulphate at 211 kg/ha + lime; (9) AN70L = ammonium nitrate at 70 kg/ha + lime; and (10) AN211L = ammonium nitrate at 211 kg/ha + lime.

### 4.3. Plant and Soil Sampling and Analysis

Plant above- and belowground biomass samples were collected in March 2021. In the middle of the plots, 0.25 × 0.25 m^2^ quadrants were created per chosen experimental plot, then all aboveground plant samples were clipped to ground level using scissors and stored in plastic pockets. The harvested grass samples were oven-dried at 60 °C for 48 h, and the dry weight was measured. The yield was calculated as the dry weight of grass per area and presented as kg/ha. Corresponding belowground biomass samples were obtained from the experimental plots using 3 soil cores to a depth of 10 cm from each plot. Cores from each plot were pooled to mitigate root distribution variability. The roots were separated from the soil by washing and sieving through a 0.5 mm sieve. Live roots were oven-dried at 60 °C to a constant weight and expressed as dry weight kg/ha. The plant tissue samples were analysed for C, N, and P content as described by [50].

Briefly, 0.125 g of milled (1 mm sieved) samples was weighed and placed into small thin foil cups and placed in a furnace for analysis. The mass of an empty beaker was pre-weighed, and 0.5 g of milled sample was added to the beaker and placed in an oven at 110 °C for 2 h. The beakers were cooled in a desiccator for 30 min and weighed. The beakers were taken into the furnace at 450 °C for 4 h. The beaker and the ashed contents were removed, cooled, and then wetted with a few drops of distilled water, and 2 mL of conc. HCI was added to each sample. The samples were evaporated slowly to dryness on a water bath in a fume cupboard with the extractor fan on. Using a Fortuna Optifix dispenser, 25 mL of a freshly prepared 1:9 HCl solution was added to each sample, and then each sample was stirred using a rubber policeman. The rod was rinsed in a beaker of distilled water between each sample. The sample was filtered through Advantech 5B 90 mm diameter filter papers into a clean rack of sample cups. The filtrate was diluted with de-ionized water at a ratio of 5:20, then the diluted solution was analysed for P on ICP-OES. Plant C and N analyses were done using a Leco CNS 2000 (LECO Corp., St. Joseph, MI, USA).

Five subsamples (0–10 cm) were collected from each plot and mixed to make a composite sample. The samples were air-dried and sieved to <2 mm, then stored in plastic jars for further analysis. Soil pH was measured using a 1:2.5 ratio of soil/distilled water as well as a 1:2.5 ratio of soil/1 M KCl. Total C and N were analysed using the LECO CNS 2000 (LECO Corp., St. Joseph, MI, USA). Liming significantly increased soil pH, while N fertilisation led to soil acidification. Soil pH in the lime-only treatment was 6.56, while it ranged from 3.27 to 4.11 in N-treated plots, compared to 4.31 in the control. Interestingly, both N and liming did not change the soil organic carbon compared to the control. Only AN211 increased the SOC compared to AN70. The SOC ranged from 49.7 to 57.6 g/kg across treatments. Microbial biomass carbon was determined via modified chloroform fumigation extraction [51]. Urease and dehydrogenase activities were measured using colorimetric assays [52].

### 4.4. Statistical Analysis

Plant and soil data were analysed using multiple statistical approaches to understand treatment effects and mechanisms. One-way ANOVA tested treatment effects on individual response variables, with Tukey’s post hoc tests used for mean comparisons (*p* < 0.05). Pearson correlation analysis was used to investigate the relationship between measured variables, while principal component analysis (PCA) was used to determine drivers of variation using the FactoMineR and factoextra packages. All statistical analyses were performed using R version 4.1.0 with significance set as *p* < 0.05.

## 5. Conclusions

This study shows how long-term N application and liming affect the biomass production, nutrient composition, and plant tissue stoichiometry of the semi-arid grassland. The results highlight that belowground plant element ratios are more responsive to treatments than aboveground ratios and may better predict soil nutrient dynamics. Results show that high N application increased plant N:P ratios beyond optimal ranges for microbial processing, while both liming and N fertilisation unexpectedly decreased root C:N ratios. Despite reduced C:N ratios and increased biomass, there was no corresponding increase in soil organic carbon, indicating a disconnection between plant productivity and C sequestration and soil pH as the dominant driver under these management regimes. The combination of lime with ammonium nitrate and ammonium sulphate showed particularly complex interactions in nutrient allocation patterns, highlighting the importance of considering both the form and rate of nitrogen application when managing grassland ecosystems for productivity and carbon sequestration. Moreover, the findings suggest that simply increasing plant biomass inputs through fertilisation or liming does not necessarily enhance C storage in semi-arid grassland, as stoichiometric and soil pH-induced constraints on decomposition processes may limit the incorporation of plant-derived C into stable soil organic matter pools. Sustainable management must balance agricultural demands with ecosystem resilience, emphasizing adaptive strategies suitable for arid and semi-arid regions. Balanced nutrient management that prevents soil acidification and maintains healthy plant–soil–microbe interactions is essential for effective carbon sequestration while sustaining grassland productivity.

## Figures and Tables

**Table 1:** Shoot, root biomass, and their ratio following long-term liming and N application.

Treatments	Shoots (kg/ha)	Roots (kg/ha)	Shoot/Root
Control	16,324 ± 1245 abc	75.8 ± 8.9 ab	19.26 ± 2.1 b
Lime	12,737 ± 987 a	259.0 ± 23.4 cd	4.92 ± 0.5 a
AS70	33,075 ± 2134 de	238.7 ± 21.3 cd	13.86 ± 1.4 ab
AS211	23,801 ± 1876 bcd	353.2 ± 29.8 d	6.74 ± 0.7 a
AN70	16,251 ± 1123 abc	247.8 ± 22.4 cd	6.56 ± 0.7 a
AN211	35,719 ± 2456 e	90.0 ± 9.8 ab	48.11 ± 4.8 c
AS70L	15,612 ± 1345 abc	148.2 ± 15.6 abc	10.53 ± 1.1 ab
AS211L	25,795 ± 1987 cde	174.6 ± 17.8 bc	14.77 ± 1.5 ab
AN70L	13,363 ± 1098 ab	38.1 ± 5.6 a	35.07 ± 3.5 c
AN211L	15,253 ± 1234 abc	104.6 ± 11.2 ab	14.58 ± 1.5 ab

## Data Availability

Data supporting this study will be made available by the corresponding author upon reasonable request.

## Supplementary Materials

The following supporting information can be downloaded at https://www.mdpi.com/article/10.3390/plants14091302/s1, Table S1: Plant elemental concentrations after long-term nitrogen and lime application; Table S2. Uptake of nitrogen, phosphorus, and potassium as affected by nitrogen and liming application; Figure S1: Correlation matrix of plant, soil, and microbial variables.
