# Supplementary material for: Long-Term Effects of Nitrogen and Lime Application on Plant–Microbial Interactions and Soil Carbon Stability in a Semi-Arid Grassland
Source: Plants (Basel). 2025 Apr 25;14(9):1302. doi: 10.3390/plants14091302 (PMC12073539; doi:10.3390/plants14091302)
Supplement: Supplementary file 1 [file plants-14-01302-s001.zip › plants-3566178-supplementary.pdf]

Supplementary Table S1. Plant elemental concentrations as affected by long-term nitrogen application and liming

Treatment	Above-ground biomass				Below-ground biomass		
	C	N	P	K	C	N	P
	%						
Control	42.40 [a]	0.8401 [abc]	0.08	1.008 [ab]	43.43	0.68 [ab]	0.071 [d]
Lime	44.31 [ab]	0.7113 [ab]	0.082	1.290 [ab]	24.99	0.63 [a]	0.061 [cd]
AS70	46.07 [bc]	0.6563 [a]	0.061	0.863 [a]	30.76	0.63 [a]	0.045 [abc]
AS211	46.97 [bc]	0.8527 [abcd]	0.065	1.096 [ab]	30.33	1.17 [bc]	0.60 [cd]
AN70	47.38 [c]	0.9243 [abcd]	0.063	1.065 [ab]	35.79	0.87 [ab]	0.035 [a]
AN211	47.55 [c]	1.1691 [d]	0.074	0.933 [a]	38.08	0.68 [ab]	0.033 [a]
AS70L	45.01 [abc]	0.7095 [ab]	0.058	1.425 [b]	37.20	0.98 [abc]	0.055 [bcd]
AS211L	46.34 [bc]	1.0132 [bcd]	0.067	0.980 [ab]	31.83	1.07 [abc]	0.042 [ab]
AN70L	45.34 [abc]	0.7996 [abc]	0.078	1.230 [ab]	41.63	1.09 [abc]	0.048 [abc]
AN211L	45.62 [bc]	1.0537 [cd]	0.066	1.155 [ab]	37.60	1.40 [c]	0.040 [ab]
**P value**	**<.001**	**<.001**	**0.073**	**0.014**	**0.173**	**<.001**	**<.001**

Supplementary Table S1. Uptake of nitrogen; phosphorus and potassium as affected by by nitrogen and liming application.

Treatment	N uptake	P uptake	K uptake
		Kg/ha	
Control	140.6 [abc]	14.35 [ab]	162.7 [a]
L	90 [a]	10.62 [ab]	166 [a]
AS70	215.4 [cd]	20.45 [bc]	284.9 [ab]
AS211	203.3 [bcd]	15.34 [ab]	261.5 [ab]
AN70	150.4 [abc]	10.14 [a]	171.5 [a]
AN211	415.7 [e]	26.55 [c]	335 [b]
AS70L	110.7 [ab]	9.09 [a]	222.6 [ab]
AS211L	262.4 [d]	17.46 [abc]	254.6 [ab]
AN70L	108.5 [ab]	10.56 [ab]	162.1 [a]
AN211L	160.2 [abc]	9.97 [a]	176.2 [a]

Values followed by a different lowercase letter in the same column are significantly different ($p < 0.05$) according to Tukey's LSD procedure. *C = control (0 kg/ha), L = lime (2250 kg/ha), AS70 = ammonium sulphate at 70 kg/ha; AS211 = ammonium sulphate at 211 kg/ha; AN70 = ammonium nitrate at 70 kg/ha; AN211 = ammonium nitrate at 211 kg/ha; AS70L = ammonium sulphate at 70 kg/ha + lime; AS211L = ammonium sulphate at 211 kg/ha + lime; AN70L = ammonium nitrate at 70 kg/ha + lime and AN211L = ammonium nitrate at 211 kg/ha + lime*

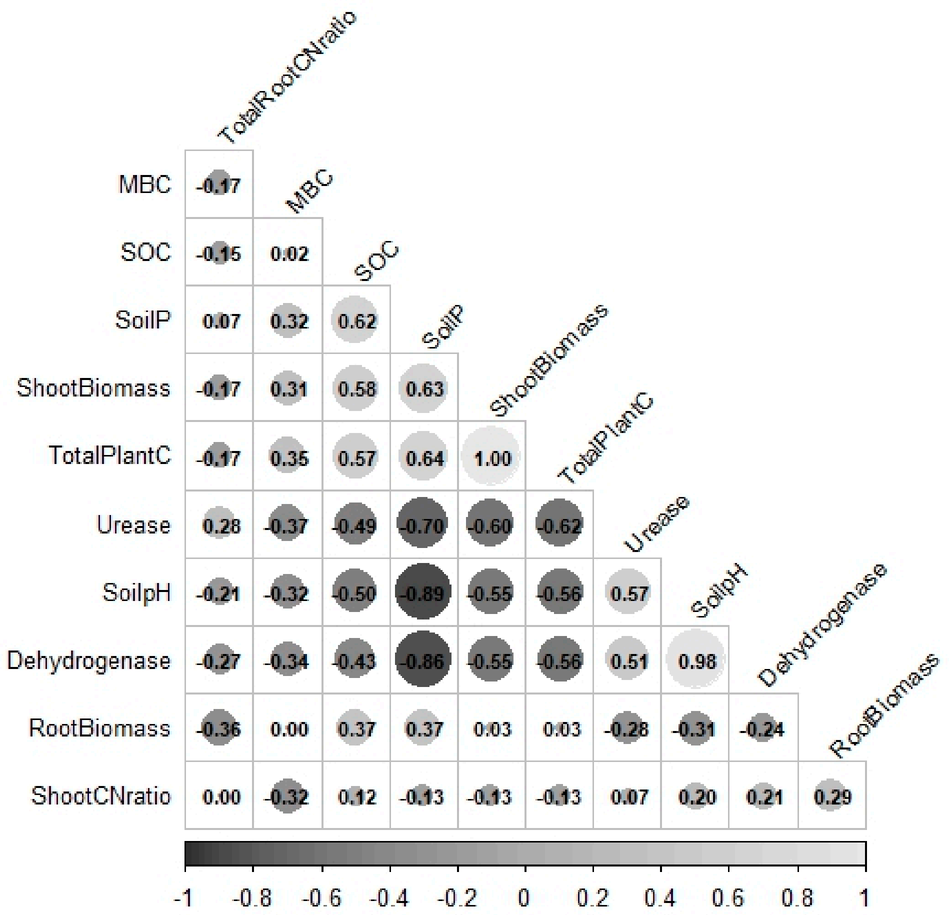

Supplementary Figure S1. Correlation matrix of plant, soil and microbial variables
